# Spontaneous abdominal wall endometriosis: A case report

**DOI:** 10.51866/cr.646

**Published:** 2025-02-26

**Authors:** Mohd Pazudin Ismail, Erinna Mohamad Zon, W Fadhlina W Adnan, Nasibah Mohamad, Nur Asma Sapiai, Sharifah Emilia Tuan Shariff

**Affiliations:** 1 MD, MMED (O&G), Department of Obstetrics & Gynaecology, School of Medical Sciences, Universiti Sains Malaysia, Jalan Raja Perempuan Zainab II, Kubang Kerian, Kelantan, Malaysia. E-mail: erinna@usm.my; 2 MD, MMED (O&G), Department of Obstetrics & Gynaecology, School of Medical Sciences, Universiti Sains Malaysia, Jalan Raja Perempuan Zainab II, Kubang Kerian, Kelantan, Malaysia.; 3 MBBCh BAO, MMED (O&G), Department of Obstetrics & Gynaecology, School of Medical Sciences, Universiti Sains Malaysia, Jalan Raja Perempuan Zainab II, Kubang Kerian, Kelantan, Malaysia.; 4 MD, MMED (Rad), Department of Radiology, School of Medical Sciences, Universiti Sains Malaysia, Jalan Raja Perempuan Zainab II, Kubang Kerian, Kelantan, Malaysia.; 5 MD, MMED (Rad), Department of Radiology, School of Medical Sciences, Universiti Sains Malaysia, Jalan Raja Perempuan Zainab II, Kubang Kerian, Kelantan, Malaysia.; 6 MD (UKM), MPATH(ANATOMIC PATHOLOGY), Department of Pathology, School of Medical Sciences, Universiti Sains Malaysia, Jalan Raja Perempuan Zainab II, Kubang Kerian, Kelantan, Malaysia.

**Keywords:** Abdominal wall endometriosis, Extrapelvic endometriosis, Abdominal wall mass

## Abstract

Abdominal wall endometriosis (AWE) is a rare type of endometriosis, with an incidence ranging from 0.1% to 0.4%. It requires a high index of suspicion to avoid delays in diagnosis and treatment. This case is rather special because AWE occurred without a history of abdominal surgery or pelvic endometriosis. Herein, we report the case of a 48-year-old para-3 woman with localised cyclical abdominal pain associated with abdominal distension. On assessment during menstruation, there were localised tenderness at the right suprapubic area and a non-tender suprapubic mass corresponding to a 14-week-sized gravid uterus. An ultrasound revealed the presence of a heterogeneous hypoechoic lesion at the subcutaneous layer of the right suprapubic region sized 1.8×3.2×4.4 cm with poor demarcation. There were also multiple uterine fibroids varying in size and location. She underwent exploratory laparotomy, total abdominal hysterectomy with bilateral salpingo-oophorectomy and abdominal wall mass resection. Intraoperatively, the right abdominal wall mass measuring 6x5 cm and involving the subcutaneous layer was found to adhere to the rectus sheath with some chocolate-stained areas without connection to the peritoneal cavity. Additionally, multiple uterine fibroids were noted. There was no pelvic endometriosis, and the other pelvic organs were normal. The histopathological diagnosis of the abdominal wall mass was endometriosis. AWE should be one of the differential diagnoses even in the absence of previous surgery when encountering a patient with an abdominal wall mass especially when it is related to the menstrual cycle.

## Introduction

Abdominal wall endometriosis (AWE) is a rare type of endometriosis. Its incidence is reported to range from 0.1% to 0.4%, but this is likely underreported because many cases go undiagnosed.^1^ AWE is more common in women who have undergone abdominal surgery, either laparoscopically or via laparotomy, and any diagnostic procedure performed through the abdomen. It is associated with a caesarean scar and hysterectomy at 57% and 11%, respectively, but about 20% of cases do not have any abdominal scar.^2^ The incidence is believed to be rising due to the increasing rate of caesarean sections. Herein, we report a rare case of spontaneous AWE without a history of abdominal surgery or pelvic endometriosis.

## Case presentation

A 48-year-old para-3 Malay woman was referred from a local clinic for worsening cyclical abdominal pain for 6 months. The pain was dull, aching and localised in the right lower abdomen. It started on day 1 of menstruation and lasted throughout her menstrual period, which was partially resolved with oral analgesia. She also noticed an abdominal mass that gradually increased in size and arose from the suprapubic area to below the umbilical level, which was associated with an increase in her menstrual blood flow. The patient experienced tenesmus, pellet-like stool, constipation and frequency. There was neither dyschezia, haematochezia, dyspareunia, dysuria nor haematuria. Her obstetrics history was uneventful, and she had three spontaneous vaginal deliveries. She had a strong family history of malignancy, where her maternal aunt passed away at the age of 40 years due to advanced ovarian cancer, while three of her cousins had breast and colon cancers.

Upon assessment on day 3 of menstruation, the patient had mild pallor but was haemodynamically stable. Her abdomen was soft, with localised tenderness at the right suprapubic region. There was no palpable mass on the tender area. A mass arising from the suprapubic area, centrally located, corresponded to a 14-week-sized gravid uterus. The mass was lobulated, had a firm consistency and was mobile from side to side. A bimanual examination revealed a normal cervix and a mass suggestive to be of uterine origin.

**Figure 1.**
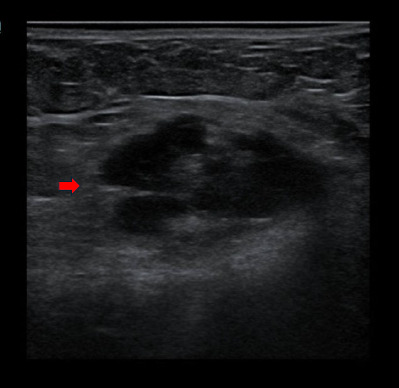
Well-defined heterogeneous hypoechoic lesion at the subcutaneous layer of the right suprapubic segion measuring 1.9×3.3×2.1 cm (red arrowX with no inteilesional or peripheral vascularity. The underlying muscle appears bulky, with a poor demarcation with the leeion. There is no obvious deeper extension.

An ultrasound was performed, which showed the presence of a heterogeneous hypoechoic lesion at the subcutaneous layer of the right suprapubic region (Figure 1).

**Figure 2.**
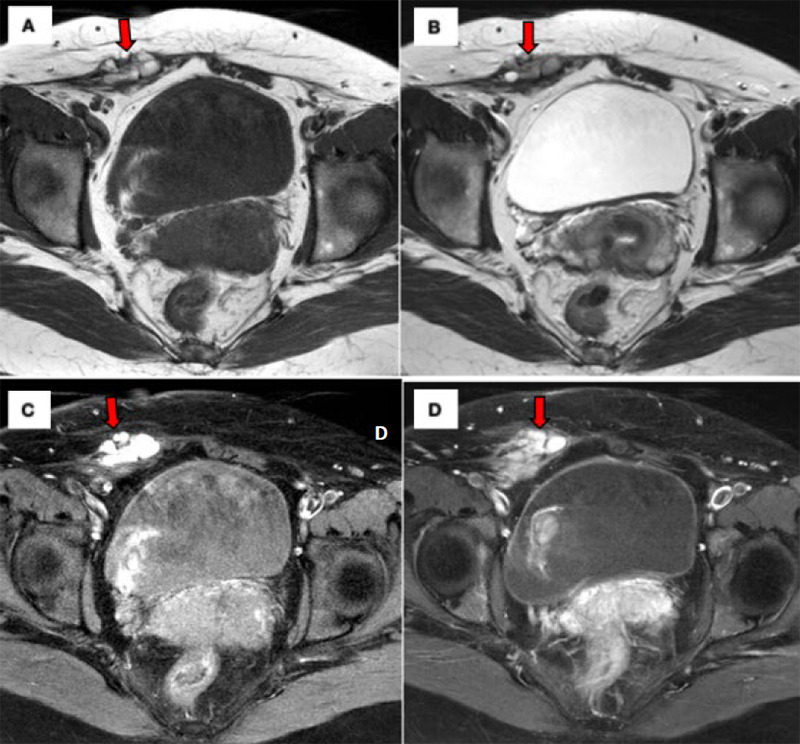
Magnetic resonance imaging of the pelvis in axial view: (A) Tl-weighted image depicts a lobulaten hyperintense lesion in the right rectn muscle. (1$) The lesion is heterogeneous and hypointense on die T2-weighted image, a characteristic known as ‘T2 shading’, and (C) hyperintense on the Tl fat-suppressed sequence, with no Oefinite enhancement in the poit-cnntrast study. (ID) The red arrow shows the right abdominal wall mass.

Multiple uterine fibroids of varying sizes were also found at the fundal and lower parts of the uterus, both anteriorly and posteriorly. The endometrial lining was regular but distorted due to the multiple fibroids. No adnexal mass was noted, and the bilateral kidneys were normal. We proceeded with magnetic resonance imaging (MRI) of the abdomen and pelvis to delineate the mass with the possibility of extrapelvic endometriosis. The magnetic resonance images of the abdomen and pelvis are shown in Figure 2.

**Figure 3.**
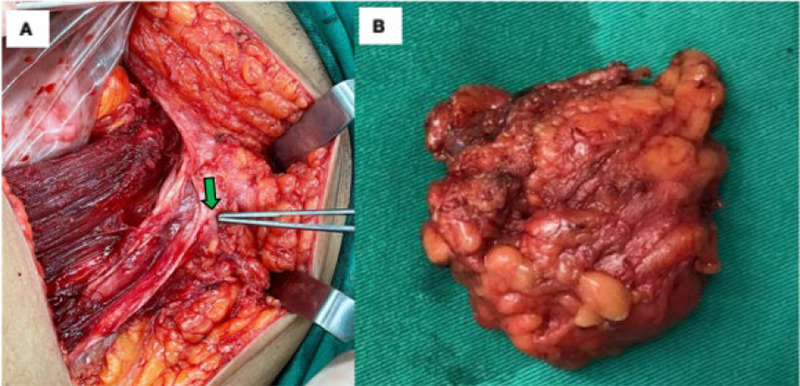
(A) The right abdominal wall mass measuring 6×5 cm at the subcutaneous layer just above the symphysis pubis adhered to the rectus sheath with some chocolate-stained areas (green arrow). No connection was seen between the mass and the peritoneal cavity. (B) Excised specimen of the right abdomi nal ‘wall mass.

**Figure 4.**
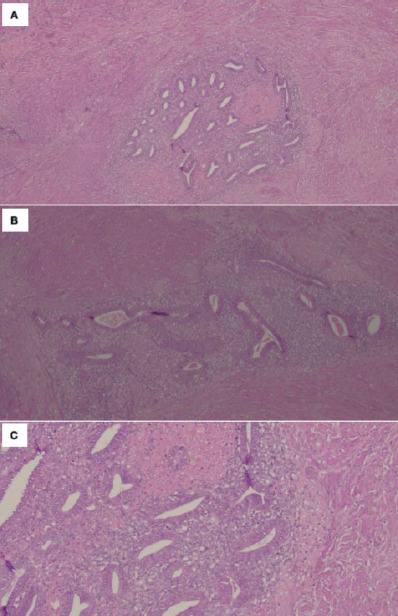
(A and B) H&E ×40: Section showing the endometriotic foci within the abdominal muscle. (C) H&E ×400: High-power-field view of endometriosis, characterised by the presence of endometrial glands and stroma.

The patient underwent exploratory laparotomy, total abdominal hysterectomy with bilateral salpingo- oophorectomy and abdominal wall mass resection. Intraoperatively, the right abdominal wall mass measuring 6×5 cm and involving the subcutaneous layer was found to adhere to the rectus sheath with some chocolate-stained areas. There was no connection seen between the mass and the peritoneal cavity, confirming that the mass was located above the rectus sheath and did not communicate through the peritoneal cavity (Figure 3). There were also multiple uterine fibroids with a normal endometrial cavity. Additionally, the ovaries and fallopian tubes were normal. There was no clinical finding suggestive of pelvic endometriosis. The histopathological diagnosis of the abdominal wall mass was endometriosis (Figure 4). The postoperative period was uneventful.

## Discussion

AWE is a rare type of endometriosis. Its incidence is reported to be higher in women who have undergone abdominal surgery, either laparoscopically or via laparotomy, or any diagnostic procedure performed through the abdomen and multiparous women. Spontaneous AWE is rare. It can present with various symptoms, and many patients initially present to surgical clinics with lumps and bumps on the abdomen.

The pathogenesis of endometriosis is still unknown; however, some theories are accepted, including retrograde menstruation, coelomic metaplasia, embryonic rest theory and lymphovascular metastasis theory. The commonest theory for AWE suggests that endometrial cells are mechanically seeded into the cutaneous wall fascia or surrounding subcutaneous tissues during surgery. However, in our case, without past abdominal surgery, coelomic metaplasia is believed to be related to spontaneous AWE. In the past decade, a stem cell-based hypothesis has emerged (among many others) to explain the pathogenesis of endometriosis, and this theory has been revised further for better understanding.^3^

The symptoms of AWE may vary among patients, but the classical triad is a history of abdominal surgery and cyclical pain associated with menstruation and nodules near the surgical scar.^4^ Cyclical bleeding is a pathognomonic sign of AWE.^5^ With the presence of an abdominal mass and heavy menstrual bleeding in our patient, the diagnosis was tricky; however, the localised cyclical pain and the sonographic finding triggered the possibility of AWE with concurrent multiple uterine fibroids. Cyclical pain should raise concerns about menstruation-related diseases, especially endometriosis (extrapelvic or ectopic). Patients may also present with lumps and bumps during visits to surgical clinics. The condition is commonly mistaken for hernia, lipoma, sebaceous cyst, haematoma, abscess and other benign or malignant abdominal wall tumours.

Ultrasound is a useful diagnostic tool for evaluating extrapelvic endometriosis and its extension. Masses are typically purely solid (67%), mixed cystic and solid (24%) and cystic with low-level internal echoes (9%).^6^ MRI is highly accurate in diagnosing extraperitoneal endometriosis. It allows the identification of lesions hindered in the adhesions or subperitoneal region. In addition, MRI is superior to other modalities, as it can depict lesions at the anterior or posterior pelvic compartment. Endometriosis is typically iso- to hyperintense on T1-weighted images. T2 shading is a hypointense signal on T2-weighted images owing to the presence of deoxyhaemoglobin and methaemoglobin. Endometriosis may demonstrate peripheral enhancement in post-contrast studies and variable restricted diffusions.^7^ Fine-needle aspiration cytology (FNAC) is an accurate, inexpensive diagnostic procedure for women with abdominal wall masses. It has been used as a diagnostic tool for AWE, but sometimes, its diagnostic use is limited due to the limited amount of sample material as well as the presence of fibrotic tissue and the controversial issue of new implants following the procedure.^8^ In this case, we did not proceed with FNAC, as the history and clinical and imaging findings were highly suggestive of AWE.

AWE is a rare condition, and the evaluation of a female patient with an abdominal wall mass should include thorough history-taking, particularly focusing on cyclical pain, bleeding and menstruation-related symptoms. Past abdominal surgery raises the possibility of AWE.

Bedside ultrasound is one of the simple imaging techniques that can be used in the primary care setting to evaluate abdominal wall masses prior to referral.

In this case, total abdominal hysterectomy with bilateral salpingo-oophorectomy and abdominal wall mass excision were conducted, as the patient had symptomatic uterine fibroids with a strong family history of malignancy and was approaching menopause. The most appropriate treatment of AWE is wide local excision with or without hormonal treatment. The optimal time for surgery is during menstruation, as lesions are more easily visualised. This timing can also help surgeons identify an appropriate surgical margin of 1 cm to reduce recurrence and the risk of malignancy.^9^ Because wide excision is the standard treatment, mesh repair may be required, especially when a large area of fascia is involved. Medical treatment is not effective as a primary treatment, but it is used following surgery to avoid recurrence and for patients with pelvic endometriosis.^10^ Both high-frequency ultrasound and cryoablation are effective in reducing the pain score and lesion size in patients with AWE; however, there are limited data on the recurrence rate.^11^ The overall outcome is good following adequate surgical resection with or without medical treatment.^2,5,9,10^

## Conclusion

- Menstruation-related symptoms should trigger the possibility of ectopic or extrapelvic endometriosis.
- The classical triad of AWE is a history of abdominal surgery and cyclical pain associated with menstruation and nodules near the surgical scar.
- AWE should be one of the differential diagnoses for women of reproductive age presenting with an abdominal wall mass.
- The absence of an abdominal scar following uterine surgery does not rule out AWE.
- In the primary care setting, maintaining a high index of suspicion for abdominal endometriosis is important to avoid delays in diagnosis and treatment. Primary care doctors need to be proficient in performing bedside ultrasound for diagnosis.
- The precise pathogenesis of AWE and endometriosis is still unclear. Thus, further research is needed to identify the exact causes.
